# Engineering Poly(Lactic-co-Glycolic Acid) (PLGA)-Based Microspheres for Controlled Corticosteroid Delivery in Intra-Articular Cartilage

**DOI:** 10.3390/pharmaceutics18070893

**Published:** 2026-07-21

**Authors:** Pamela Rose V. Samonte, Noelle K. Comolli

**Affiliations:** Department of Chemical and Biological Engineering, Villanova University, 800 E. Lancaster Ave., Villanova, PA 19085, USA

**Keywords:** PLGA microspheres, osteoarthritis, intra-articular drug delivery, controlled release, corticosteroid stability, ester hydrolysis

## Abstract

**Background**: Osteoarthritis (OA), affecting approximately 240 million people worldwide, currently lacks targeted, long-acting therapeutic options. This study investigates how physicochemical properties (i.e., size, surface charge, polydispersity) influence poly(lactic-co-glycolic acid) (PLGA) microsphere (MS) diffusion into articular cartilage for enhanced corticosteroid delivery. **Methods**: PLGA MSs were synthesized via oil/water emulsions to create a variety of sizes and surface charges. MSs were loaded with corticosteroid (H-17-B) and release kinetics were studied in vitro and analyzed via HPLC. Diffusion of the MSs was investigated via a bovine explant model. **Results**: Unmodified PLGA MSs (approximately 0.58–0.98 µm) were synthesized via single-stage oil/water emulsion with varying poly(vinyl alcohol) concentrations and sonication intensities. All formulations exhibited near-neutral surface charges (−0.27 to 4.28 mV). Smaller particles achieved greater cartilage penetration, with bi-exponential diffusion models (R^2^ = 0.706–0.999) outperforming classical Fickian approaches. However, multi-timepoint validation demonstrated fundamentally non-diffusive transport, likely governed by steric exclusion from the dense collagen network (0.05–0.06 µm pore size). Surface functionalization with avidin/palmitic acid or polyethylene glycol (PEG)/biotin yielded microspheres with controlled properties (0.36–0.97 µm; PDI: 0.10–0.32). In vitro release studies with hydrocortisone-17-butyrate (H-17-B; encapsulation efficiency of 79.8% ± 4.8%) demonstrated biphasic kinetics best fit by bi-exponential models (R^2^ > 0.95). Unmodified microspheres exhibited 7.1% cumulative release by Day 14. High-performance liquid chromatography revealed that H-17-B undergoes ester hydrolysis to hydrocortisone during release, with surface modifications significantly affecting drug stability. Specifically, PEGylated microspheres maintained 96% of the drug in H-17-B form at Day 14 compared to only 37% for unmodified particles. Release was governed by PLGA degradation with concentration-independent kinetics, enabling predictable dose scalability. **Conclusions**: This work establishes that the behavior of PLGA microspheres in cartilage is controlled by size, charge, and surface functionalization. Surface modifications overcome physical barriers while stabilizing the encapsulated corticosteroid against premature hydrolysis, providing a framework for designing intra-articular drug delivery systems for osteoarthritis treatment.

## 1. Introduction

Osteoarthritis (OA) affects over 240 million people worldwide, causing progressive joint degeneration and disability, particularly in individuals over the age of 60 [1,2,3,4,5]. Pathologic changes in the cartilage, bone, ligaments, and synovium can result from multiple factors, including joint injury, obesity, aging, and heredity, which can lead to joint dysfunction and functional limitation [1,2,3]. Articular cartilage (AC) is an avascular, aneural tissue composed primarily of water (65–80%), extracellular matrix (ECM), and chondrocytes [2]. The ECM mainly comprises a dense collagen fibril network and highly negatively charged proteoglycans with glycosaminoglycan (GAG) side chains. This dense, charged matrix creates a significant barrier to drug penetration, with collagen pore sizes of 0.06–0.2 µm and GAG chain spacing of 0.002–0.006 µm limiting delivery of therapeutic agents [6,7]. Cartilage degeneration involves chondrocyte apoptosis and ECM degradation, which can aggravate inflammation and ultimately accelerate OA progression [2,8].

OA is an irreversible chronic disease, and current treatments, which include physical therapy, pharmacological interventions, and surgical procedures, mainly focus on symptom management rather than disease modification [9,10]. These approaches only provide temporary pain relief (days to weeks) and require frequent re-administration [8,9,11]. The lack of sustained, targeted drug delivery to deep cartilage tissue represents a critical unmet need in OA therapy. Nano- and microparticle drug delivery systems (DDSs) have emerged as promising platforms for targeted therapeutic delivery, improving treatment specificity while reducing dosing frequency and systemic exposure [12,13,14]. These systems can be engineered with specific physicochemical properties to enhance tissue accumulation and therapeutic efficacy.

Current intra-articular therapies for osteoarthritis, including corticosteroids (hydrocortisone-17-butyrate, dexamethasone, triamcinolone acetonide) and hyaluronic acid, provide only temporary symptom relief due to rapid clearance from the joint space (half-lives of 4–8 h to 1–3 days) [8,9,11]. To overcome these limitations, particulate delivery systems have been investigated. PLGA-based microspheres have demonstrated clinical success, with Zilretta (triamcinolone acetonide extended-release injectable suspension) representing the first FDA-approved PLGA microsphere formulation for sustained intra-articular corticosteroid delivery [15,16]. Additional systems under investigation include PLGA nanoparticles, liposomal formulations, chitosan particles, and injectable hydrogels [5,17,18,19]. While PLGA systems offer biodegradability and tunable release, systematic investigation of how surface functionalization affects the interplay between tissue penetration, release kinetics, and drug stability in the intra-articular environment remains limited.

Intra-articular drug delivery faces dual challenges: rapid clearance from synovial fluid and/or synovium and limited penetration into dense cartilage ECM [17,19]. The tight packing of collagen fibrils and negatively charged GAG chains creates both steric and electrostatic barriers [6,7]. Nanoparticles with sizes <0.015 µm can penetrate the full thickness of the cartilage, while those with sizes of 0.015 to 0.06 µm reach only superficial zones [5,6]. These size and charge limitations hinder efficient drug delivery and adsorption, ultimately resulting in lower drug half-lives (i.e., 0.1–6 h) and retention times (i.e., 6–25 d) [6]. Systematic investigation of particle size and surface properties is needed to optimize deep cartilage penetration for sustained drug delivery.

Despite these advances, quantitative understanding of how surface modification affects PLGA microsphere penetration through cartilage remains limited. This study systematically investigates the effect of surface modification (unmodified, avidin, PEG) on PLGA microsphere cartilage penetration, examining formulations with varying particle sizes, surface charges, and polydispersity indexes to: (1) quantify cartilage penetration using bi-exponential diffusion modeling, (2) correlate surface properties with transport behavior, and (3) identify optimal formulations for sustained intra-articular drug delivery to deep cartilage zones.

## 2. Materials and Methods

### 2.1. Materials

PolySciences (Warrington, PA, USA): poly(lactic-co-glycolic acid) (PLGA; 50:50 lactide:glycolide; IV 0.6 dL/g), poly(vinyl alcohol) (PVA; 88% hydrolyzed), methoxy poly(ethylene glycol) amine (mPEG-NH_2_; Mp 2000); ThermoScientific (Waltham, MA): dichloromethane (DCM; ACS reagent grade, ≥99.5%), 1,1-dioctadecyl-3,3,3′,3’-tetramethylindocarbocyanine perchlorate (DiI, DiIC_18_(3)), Pierce^TM^ Biotin Quantification Kit; Sigma-Aldrich (St. Louis, MO, USA): Sodium deoxycholate (≥97%), palmitic acid *N*-hydroxysuccinimide ester (NHS–palmitic acid; ≥98%), phosphate-buffered saline (1× PBS, pH 7.4), HABA (4’-hydroxyazobenzene-2-carboxylic acid, hydrocortisone-17-butyrate, hydrocortisone; ApexBio Technology (Houston, TX, USA): Sulfo-NHS-biotin (sulfosuccinimidyl 6-(biotinamido) hexanoate, 100 mg); Spectrum Laboratories (Piscataway, NJ, USA): Spectra/Por Dialysis Membrane Kit (3500 Dalton, 18 mm flat width); Lee Biosolutions (now Medix Biochemica; Maryland Heights, MO, USA): Avidin, 500 mg; Vector Laboratories (Newark, CA, USA): VECTASHIELD^®^ Antifade Mounting Medium with DAPI (10 mL). Hydrocortisone-17-butyrate and hydrocortisone were used as received.

### 2.2. PLGA Microsphere Preparation

PLGA microspheres were generated using a single-stage oil/water emulsion following a previously reported method with modifications [20]. Briefly, 39 mg/mL of 50:50 (lactide:glycolide) PLGA was dissolved in DCM and sonicated (Branson Digital Sonifier, Danbury, CT, USA) at 20% to fully homogenize the oil phase. For drug-loaded microspheres, hydrocortisone-17-butyrate (H-17-B) in DCM was added to achieve mass ratios of 0.1, 0.2, or 0.3 (*w*/*w*) H-17-B relative to PLGA prior to emulsification. The oil phase was then rapidly combined with PVA (1–5 wt%) at an oil-to-water ratio of 5:14 in a separate borosilicate glass container. The generated oil/water emulsion was then sonicated (20–30%) for 120 s on ice. The emulsion was allowed to stir for at least 12 h at 200 rpm, 25 °C, to allow evaporation of the organic solvent. Following solvent evaporation, the emulsion was resuspended and washed three times with DI water and centrifuged for 12 min at 12,000 *g*. Following the third wash, the MSs were resuspended in DI and flash-frozen with N_2_ (*l*) and lyophilized (Labconco Freeze Dry System, Kansas City, MO, USA) at −80.5 °C, 0.062 mbar. All generated MS formulations were generated in triplicate and were stored at −20 °C in darkness until further use.

### 2.3. Surface Modification

#### 2.3.1. Avidin/Palmitic Acid Conjugate Gel Preparation

Avidin (10 mg/mL) was reacted with 10-molar excess of NHS–palmitic acid in PBS solution with 2% (*w*/*v*) deoxycholate over 12 h with gentle agitation at 37 °C. Following the reaction, excess fatty acid and hydrolyzed ester were removed via dialysis (MW_cutoff_ = 3.5K) against a 0.15% (*w*/*v*) deoxycholate for 72 h with light agitation. The final product was stored at 4 °C until further use. The avidin concentration in the final conjugate gel was quantified using a direct HABA binding assay. HABA (4′-hydroxyazobenzene-2-carboxylic acid; Sigma-Aldrich) was dissolved in 2% (*w*/*v*) deoxycholate/PBS buffer and mixed with the avidin conjugate sample. The formation of the HABA–avidin complex was measured spectrophotometrically at 500 nm, and avidin concentration was calculated using a standard curve of known avidin concentrations (0–10 mg/mL) prepared under identical conditions.

#### 2.3.2. Poly(Ethylene Glycol)/Biotin Conjugate Gel Preparation

Methoxy poly(ethylene glycol) amine (mPEG-NH_2_; 20 mg/mL) was first dissolved in PBS, from which sulfosuccinimidyl 6-(biotinamido) hexanoate (sulfo-NHS-biotin) was added at a 20-molar excess. The solution was stirred at ambient temperature for 4 h. Following the reaction, the solution was further purified via dialysis (MW_cutoff_ = 3.5K) against pure PBS. The final conjugate gel was stored at 4 °C for future use. The presence of biotin was verified using the Pierce Biotin Quantification Kit (ThermoFisher Scientific, Waltham, MA, USA).

#### 2.3.3. Avidinated PLGA MS Preparation

Avidinated PLGA MSs were generated through a slight modification of the original single-stage oil/water emulsion method. Briefly, 2 mL of the avidin/palmitic acid conjugate gel was first added to the water phase, from which the solution was vortexed for 30 s prior to use. The oil and water phases were then combined following the protocol detailed in Section 2.2. Functionalization of avidinated microspheres was verified using a direct HABA binding assay detailed in Section 2.3.1.

#### 2.3.4. PEGylated PLGA MS Preparation

PLGA microspheres were PEGylated at a ratio of 500 µg of biotin–PEG conjugate gel per 1 mg of MS. The conjugate gel–MS mixture was then incubated at 37 °C, 220 rpm in darkness for 20 min. The PEGylated MSs were then washed three times with sterile DI and lyophilized as described in Section 2.2. Functionalization of PEGylated microspheres was verified using the Pierce Biotin Quantification Kit (ThermoFisher Scientific, Waltham, MA, USA).

#### 2.3.5. Fluorescent Labeling

Fluorescent PLGA microspheres were generated by incorporating DiI (DiIC_18_(3)) into the oil phase during fabrication. DiI was dissolved in DCM at a concentration of 10 mg/mL and added to the PLGA/DCM at a ratio of 2.5 µL DiI/1 mL DCM. The fluorescently labeled microspheres were then fabricated following the standard emulsion procedure described in Section 2.2.

### 2.4. Physicochemical Characterization

#### 2.4.1. Dynamic Light Scattering

Particle size distribution, polydispersity index, and zeta potential were measured using dynamic light scattering (DLS; NanoBrook Omni particle size and zeta potential analyzer, Brookhaven Instruments, Holtsville, NY, USA). Microspheres were suspended in PBS (1×, pH 7.4) at a concentration of 0.15 mg PLGA/2 mL PBS. Size measurements were performed at 25 °C with a detection angle of 90°. Zeta potential measurements were conducted in PBS to assess surface charge. All measurements were performed in triplicate.

#### 2.4.2. Scanning Electron Microscopy

Scanning electron microscopy (SEM; Hitachi S-4800, Tokyo, Japan) was employed to observe microsphere morphology. Microsphere samples were staged on double-sided carbon tape and sputter-coated with Au. An accelerated voltage of 10 kV was employed for SEM imaging. All SEM post-imaging processing was completed using ImageJ. Particle size distributions were analyzed by measuring the Feret’s statistical diameter of at least 500 microspheres in the SEM image. The Feret diameter is calculated from the perpendicular distance between parallel tangents on opposite sides of spherical profiles [21]. The diameters were distributed across up to 17 bins (i.e., 0.0, 0.25, 0.5, …, 3.75, 4.0 µm), plotted with respect to relative frequency (%), and fitted against a non-linear Gaussian distribution to determine the average particle size (mean ± standard deviation).

#### 2.4.3. Fluorescence Microscopy

For fluorescent microsphere diffusion analysis into osteochondral plugs, sagittal sections of the cartilage plugs were gently rinsed, surgically separated from the cortical bone, embedded in Tissue-Tek O.C.T. Compound (Sakura, Torrance, CA, USA), and cryosectioned longitudinally through the cartilage at a thickness of 5 µm. The cryosectioned samples were mounted with VECTASHIELD^®^ Antifade Mounting Medium with DAPI, covered with a glass coverslip, and sealed with clear nail polish. Sections were observed using an Invitrogen EVOS M700 imaging system (ThermoFisher Scientific, Waltham, MA, USA) to visualize DiI-labeled microsphere penetration. The embedded samples and slides were stored at −80 °C and 4 °C, respectively, in darkness until further use. All fluorescent post-imaging processing was completed using either Fiji version 1.54p or ImageJ version 1.54g.

### 2.5. Cartilage Penetration Studies

Bovine osteochondral plugs (aged 24–36 months) were obtained from frozen, healthy mature hind limbs (Animal Technologies Inc., Tyler, TX, USA). Cylindrical plugs (diameter: 6.25 mm) were extracted using a coring drill and sectioned to expose fresh articular cartilage surfaces. Fluorescently labeled microspheres (DiI-loaded) were suspended in PBS at 10 mg/mL and introduced to the articular surface. The plugs were incubated at 25 °C for 1–24 h to allow microsphere penetration. Following incubation, the sagittal sections of the cartilage plugs were gently rinsed, surgically separated from the cortical bone, embedded in Tissue-Tek O.C.T. Compound (Sakura, Torrance, CA, USA), and cryosectioned longitudinally through the cartilage at a thickness of 5 µm. The cryosectioned samples were stained with VECTASHIELD^®^ Antifade Mounting Medium with DAPI to minimize drying, covered with a glass coverslip, and sealed with clear nail polish. The sections were observed using an Invitrogen EVOS M700 imaging system (ThermoFisher Scientific, Waltham, MA, USA). Fluorescence intensity profiles were quantified as a function of tissue depth using MATLAB. Approximately 8 profiles per section (oriented in either the x- or y-direction) were analyzed at a spacing between 385 and 548 µm and averaged for each sample. Data represent mean ± SD from *n* = 3 samples per formulation and timepoint.

### 2.6. Mathematical Modeling

#### 2.6.1. Cartilage Diffusion Analysis

Microsphere penetration into cartilage was modeled using both mono-exponential (Fickian) and bi-exponential diffusion models. The bi-exponential model is given by Equation (1):

(1) $$C\left(x,t\right)=A_{1}\cdot erfc\left(\frac{x}{2\sqrt{D_{1}t}}\right)+A_{2}\cdot erfc\left(\frac{x}{2\sqrt{D_{2}t}}\right)$$

where *C*(*x*,*t*) is the concentration at depth *x* (µm) and time *t* (h), *A*_1_ + *A*_2_ = 1 (normalized fractions), *D*_1_ is the fast diffusion coefficient (also known as *D*_fast_; µm^2^/s), *D*_2_ is the slow diffusion coefficient (also known as *D*_slow_; µm^2^/s), and *erfc* is the complementary error function. Model fitting was performed using MATLAB (R2025a) with non-linear least-squares optimization. Both single-timepoint analysis (24 h) and multi-timepoint validation (1–24 h) were conducted to assess model validity.

#### 2.6.2. Drug Release Kinetics

Drug release profiles were fitted to multiple kinetic models, including Fickian, Higuchi, bi-exponential, Weibull, and first-order models. The bi-exponential release model is given by Equation (2):

(2) $$\frac{M_{t}}{M_{\infty }}=\varphi_{fast}\left(1-exp^{-k_{fast}t}\right)+\varphi_{slow}\left(1-exp^{-k_{slow}t}\right)$$

where *M_t_* and *M_∞_* define the respective cumulative amounts of released drug at time *t* (d) and at infinite time, respectively, *ϕ*_fast_ + *ϕ*_slow_ are the fractions of drug released during the fast and slow phases, respectively (*ϕ*_fast_ + *ϕ*_slow_ = 100%), and *k*_fast_ and *k*_slow_ are the respective rate constants for each phase [22,23]. Model selection was based on R^2^ values and residual analysis.

### 2.7. Drug Release Studies

Drug loading was verified by dissolving microspheres (3 mg/mL) in acetonitrile and quantifying H-17-B concentration by high-performance liquid chromatography (HPLC). Encapsulation efficiency (EE%) was calculated as the ratio of the mass of encapsulated and surface-bound drug with respect to the mass of initial drug added [EE% = (*M*_encapsulated+surface-bound drug_)/*M*_initial drug added_ × 100%]. Drug release studies with H-17-B-loaded PLGA microspheres were performed using a Hanson SR-8 Plus Cell Dissolution System following United States Pharmacopeia (USP) Type II dissolution standards at 50 rpm and 37 °C to simulate physiological conditions [24]. In each reaction vessel, drug-loaded PLGA microspheres were suspended in 1 mL of 1× PBS (10 mM, pH 7.4) to achieve theoretical H-17-B concentrations of 24 or 50 µg/mL, transferred to approximately 5–6 cm of dialysis tubing (MW_cutoff_ = 3.5K), and sealed. The dialysis bags were then immersed in 1× PBS dissolution medium maintained at 37 °C with continuous stirring at 50 rpm. At predetermined time intervals (1 h, 2 h, …, 336 h), 1 mL aliquots of the dissolution medium were withdrawn and replaced with fresh PBS to maintain sink conditions. The collected samples were filtered through 0.22 µm membranes and analyzed via HPLC to quantify both H-17-B and hydrocortisone (HC) concentrations. Cumulative drug release was calculated as a percentage of the initial encapsulated drug amount, with all measurements performed in triplicate.

### 2.8. High-Performance Liquid Chromatography (HPLC)

Hydrocortisone-17-butyrate (H-17-B) and hydrocortisone (HC) were quantified via high-performance liquid chromatography (HPLC; Shimadzu HPLC with 20AB pump and 20AC autosampler, Baltimore, MD) with a Premier C18 column at a flow rate of 1.5 mL/min using a 50:50 acetonitrile (ACN):deionized water (DI) mobile phase at a λ_detection_ of 254 nm and an injection volume of 20 µL. Elution times for H-17-B were approximately 1.8 min for samples prepared in acetonitrile (encapsulation efficiency measurements) or 2.02 min for samples prepared in PBS buffer matrix (drug release measurements), while HC eluted at 1.2 min. The longer retention time observed for PBS-prepared samples is attributed to the injection solvent effect, where the aqueous sample matrix is weaker than the 50:50 ACN:DI mobile phase, causing a slight delay in analyte migration through the column. Standard curves were prepared in the corresponding solvent matrix for each measurement type to ensure accurate quantification. All HPLC samples were filtered through 0.22 µm membranes and transferred to amber vials for HPLC analysis.

### 2.9. Statistical Analysis

Data are presented as mean ± standard deviation. Two-way analyses of variance (ANOVA) followed by a Tukey post hoc multiple comparison test were applied to analyze the microsphere size, polydispersity index (PDI), surface charge, and drug release data [25]. *p*-values less than 0.05 were considered significant. All statistical analyses were performed using GraphPad Prism10.6 (GraphPad Software Inc., San Diego, CA, USA).

## 3. Results and Discussion

### 3.1. Microsphere Fabrication and Characterization

**Figure 1:**
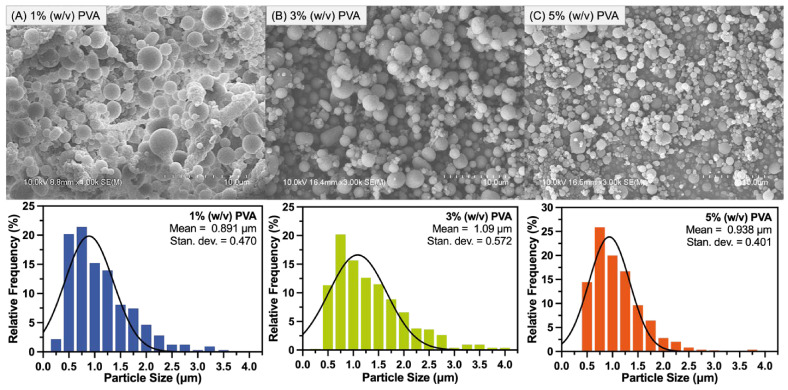
Scanning electron microscopy (SEM) images of unmodified PLGA generated at a sonication intensity of 20% with (**A**) 1% (*w*/*v*), (**B**) 3% (*w*/*v*), and (**C**) 5% (*w*/*v*) PVA. All microspheres were captured at either 3k (3%, 5% PVA) or 4k (1% PVA) magnification, with a consistent 10 µm scale bar across all panels, and were coated with up to two layers of 6 nm Au prior to SEM imaging. The non-linear Gaussian particle size distribution of each image was determined from the Feret’s diameter of at least 500 microspheres.

**Figure 2:**
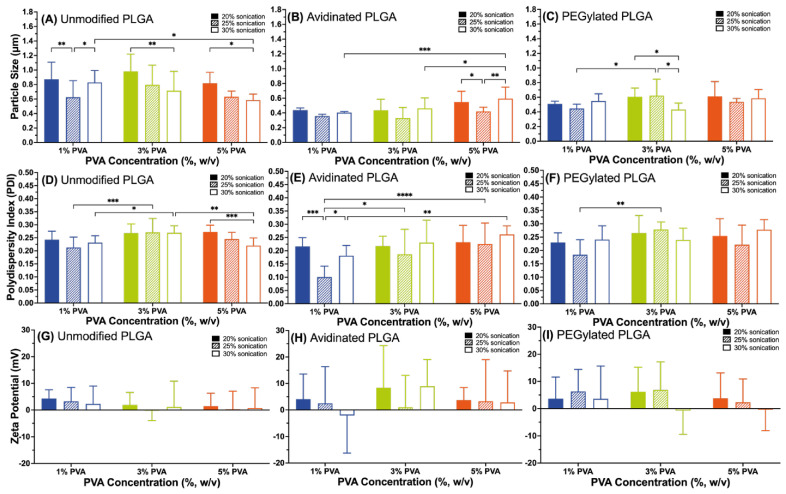
Physicochemical characteristics of unmodified, avidinated, and PEGylated poly(lactic-co-glycolic acid) (PLGA) microspheres formulated at increasing PVA concentrations (1%, 3%, and 5% (*w*/*v*) PVA in dark blue, green, and orange, respectively): (**A**–**C**) particle size, (**D**–**F**) polydispersity index (PDI), and (**G**–**I**) zeta potential. Data represent mean ± SD (*n* = 4 for all groups). Statistical significance was determined by two-way ANOVA followed by the Tukey post hoc multiple comparison test. * *p* < 0.05, ** *p* < 0.01, *** *p* < 0.001, **** *p* < 0.0001.

Unmodified PLGA microspheres of different sizes were generated by systematically varying poly(vinyl alcohol) (PVA; 1–5% (*w*/*v*)) and sonication intensity (20%, 25%, 30%) during synthesis. All unmodified MSs exhibited a smooth surface morphology, as evinced by the scanning electron microscopy (SEM) in Figure 1. Size distribution analyses revealed that increasing the PVA concentration from 1% to 5% generated up to 1.4-fold smaller particle sizes and 1.1-fold lower polydispersity indexes (PDI), with the most pronounced effects observed at 30% sonication (Figure 2). Similar to previous studies, the decreased microsphere size and PDI were likely due to changes in emulsion stability at higher PVA concentrations [26,27,28,29,30,31]. At PVA concentrations above 2.5% (*w*/*v*), increased surfactant density at the oil/water interface creates a denser protective layer around forming droplets, and higher hydrophobic acetate groups may boost the PVA surfactant activity, thus lowering surface tension and improving emulsification efficiency [26,29,32].

Zeta potential decreased as PVA concentration increased, with an up to 11.6-fold reduction observed at 25% sonication intensity (i.e., from 3.24 mV with 1% (*w*/*v*) PVA to 0.28 mV with 5% (*w*/*v*) PVA), which is in agreement with previous studies [26,29,31]. The decrease in zeta potential may be attributed to the presence of residual PVA on the MS surface, which masks any charged groups already present on the surface, particularly the methyl esters of the polymer, with PVA acetate groups [29,33]. All PVA concentrations exhibited zeta potentials that were close to neutrality, as previously observed [34], which is defined by zeta potentials between −30 mV and +30 mV [35,36,37]. Although zeta potential measurements closer to zero are generally correlated to a higher degree of instability, all microspheres exhibited PDI values of less than 0.3, which has been deemed acceptable for drug delivery applications [38,39].

The effects of sonication intensity were also monitored at the three PVA concentrations, wherein higher PVA concentrations exhibited progressively larger decreases in particle size: a 1.05-fold (1% PVA), 1.25-fold (3% PVA; *p* < 0.01), and 1.40-fold decrease (5% PVA; *p* < 0.05) as sonication intensity increased from 20% to 30% (Figure 2A). Examining sonication effects on PDI, increasing the sonication intensity significantly decreased the PDI of the microspheres, most notably at the highest PVA concentration of 5% (*w*/*v*) (1.12-fold decrease; *p* < 0.001) (Figure 2D). These observations align with previous studies showing that increased sonication energy transfer and cavitation phenomena generate elevated shear forces that more uniformly disperse emulsion droplets, resulting in both smaller particle sizes and lower PDI values [30,37,40]. With increasing sonication intensity from 20% to 30%, zeta potential showed numerical decreases at all PVA concentrations (i.e., 1.6-fold at 1% PVA, 1.9-fold at 3% PVA, and 1.8-fold at 5% PVA), though these differences did not reach statistical significance (Figure 2G). All samples exhibited near-neutral zeta potentials (−0.27 to 4.28 mV), typical of end-capped PLGA [41,42].

Surface functionalization with avidin and PEG was successfully achieved and verified. Avidin conjugation to palmitic acid via NHS ester chemistry yielded a conjugate gel with 62% avidin recovery (6.23 ± 0.12 mg/mL) as quantified by HABA dye displacement assay, with 4.2 ± 0.3 mmol biotin/mg microsphere. PEGylation was optimized at a 20-molar excess of sulfo-NHS-biotin relative to mPEG-NH_2_, demonstrating 79–97% adsorption efficiency with 38.7 ± 2.1 nmol biotin/mg microsphere. Similar to unmodified microspheres, physicochemical characterization of modified microspheres was performed across varying PVA concentrations and sonication intensities. Both avidinated and PEGylated microspheres demonstrated size-dependent responses to fabrication parameters. Avidinated microspheres showed particle sizes ranging from 0.36 ± 0.02 µm to 0.59 ± 0.16 µm across all conditions (Figure 2B). Higher sonication intensities generally produced smaller particles, with the most pronounced effect observed at 5% PVA, where particle size decreased 1.30-fold from 20% to 25% sonication (0.55 ± 0.15 µm to 0.42 ± 0.06 µm, *p* < 0.05). However, the effect plateaued at higher intensities, with minimal change or slight increase between 25% and 30% sonication, suggesting optimal performance at 25% sonication intensity, particularly at elevated PVA concentrations, where enhanced stabilization may further limit size reduction [22,43]. PEGylated microspheres (Figure 2C) exhibited similar size ranges (0.43 ± 0.09 µm to 0.62 ± 0.22 µm) with more consistent trends across PVA concentrations. The most significant reductions were at 3% PVA (a 1.4-fold decrease from both 20% to 30% and 20% to 25% sonication, *p* < 0.05). Notably, at 30% sonication, all three PVA concentrations produced statistically similar particle sizes (0.43–0.59 µm), suggesting that high sonication intensity can overcome PVA concentration effects [40,44]. Relative to unmodified microspheres (0.58 ± 0.02 µm at 5% PVA, 30% sonication), avidin conjugation and PEGylation exhibited similar sizes (0.59 ± 0.16 µm and 0.59 ± 0.12 µm, respectively).

PDI for avidinated microspheres (Figure 2E) ranged from 0.10 ± 0.04 to 0.26 ± 0.03, indicating relatively monodisperse populations. PDI generally decreased with increasing sonication intensity, with the most pronounced improvement at 1% PVA (0.22 ± 0.03 at 20% to 0.10 ± 0.04 at 25%, *p* < 0.001). The lowest PDI values were consistently achieved at 25% sonication across all PVA concentrations (0.10–0.23). PEGylated microspheres (Figure 2F) demonstrated similar PDI ranges (0.18 ± 0.06 to 0.29 ± 0.03), with higher sonication intensities yielding lower PDI values at all PVA concentrations. All surface-modified formulations maintained PDI values below 0.32, which is deemed acceptable for drug delivery applications [38,39]. Zeta potential measurements revealed that avidinated microspheres exhibited near-neutral to slightly positive surface charges (−2.09 ± 14.15 mV to 8.93 ± 10.14 mV) (Figure 2H) with considerable variability across conditions. This near-neutral charge profile is expected as the positively charged avidin protein (pI ~ 10) interacts with the negatively charged PLGA surface, resulting in charge neutralization [45]. PEGylated microspheres presented slightly positive to near-neutral zeta potentials (−0.72 ± 8.69 mV to 6.89 ± 10.30 mV) (Figure 2I), with a tendency toward more negative values at higher PVA concentrations, following the same trend as unmodified microspheres. This is characteristic of PEGylated systems, where the hydrophilic PEG layer shields the underlying charged PLGA surface [46,47], with the PVA concentration effect due to residual PVA acetate groups on the surface. The near-neutral surface charges observed for both formulations, while different from the typical negative charges of unmodified PLGA microspheres [48], confirm successful surface modification. The high variability in zeta potential measurements likely reflects the dynamic nature of surface-modified particle suspensions, where surface groups may adopt different conformations in aqueous environments [26].

### 3.2. Two-Dimensional Cartilage Diffusion

**Figure 3:**
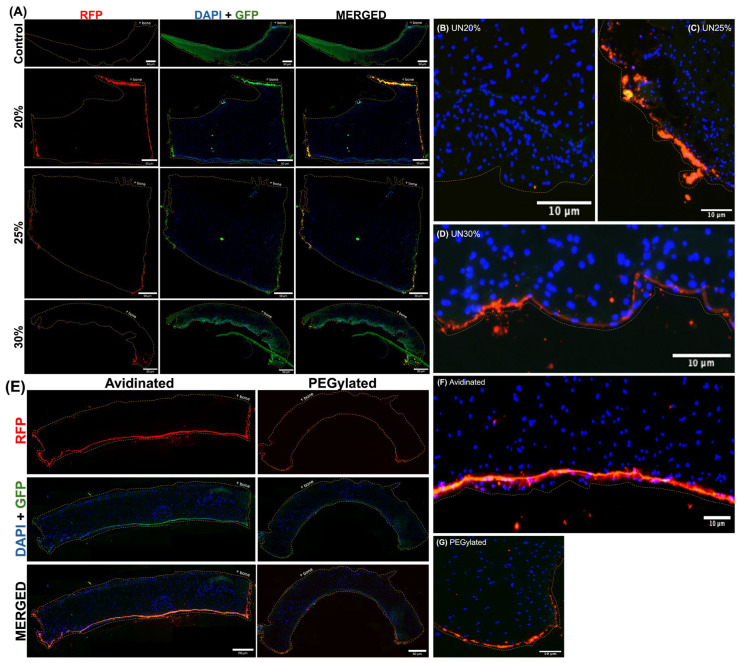
Representative micrographs of fluorescently labeled (**A**) unmodified (20%, 25%, 30% sonication intensity), (**E**) avidinated (30% sonication), and PEGylated (30% sonication) microspheres after 24 h. Close-up images of cartilage sections where diffusion of (**B**–**D**) unmodified, (**F**) avidinated, and (**G**) PEGylated microspheres was observed. Scale bars represent either 10 or 50 µm. Fluorescent channels: microspheres (red, RFP), cell nuclei (blue, DAPI), and cartilage matrix (green, GFP).

Two-dimensional diffusion of fluorescence-labeled microspheres (DiI) demonstrated that both particle size and surface chemistry significantly influence tissue penetration depth. A striking hierarchy emerged: unmodified PLGA microspheres showed limited penetration (Figure 3A–C), followed by avidinated microspheres (Figure 3E,F), and PEGylated microspheres exhibiting the deepest penetration (Figure 3E,G), representing a 2- to 3-fold improvement over unmodified formulations.

Mathematical modeling employed two complementary approaches: (1) single-timepoint analysis at 24 h using multiple diffusion models (i.e., Fickian diffusion, bi-exponential diffusion, stretched exponential, and anomalous diffusion) to characterize spatial concentration profiles, and (2) multi-timepoint validation across 1–24 h to assess whether extracted diffusion coefficients remain constant over time. The bi-exponential model (Equation (2)) provided better fits compared to classical Fickian models at individual timepoints for all three formulations (Table S1). For unmodified PLGA microspheres, single-timepoint (24 h) bi-exponential R^2^ values in the x-direction were 0.986 (20%), 0.945 (25%), and 0.978 (30% sonication), which substantially exceeded the Fickian model fits (R^2^ = 0.886, 0.765, and 0.610, respectively). The bi-exponential model extracted diffusion coefficient approximations of *D*_fast_ = 333.9–362.0 µm^2^/s and *D*_slow_ = 0.14–0.19 µm^2^/s, with the fast component representing 26.5–38.1% of transport. For the y-direction, *D*_fast_ ranged from 345.9 to 500.0 µm^2^/s with similar fast/slow fractions (17.1–37.5% fast, 62.5–82.9% slow). However, multi-timepoint validation demonstrated that unmodified microspheres do not undergo classical diffusive transport, with overall R^2^ values of −0.28094 to 0.3101 for the x-direction and −2.3579 to 0.02657 for the y-direction across sonication intensities.

The better fit observed with the bi-exponential model at individual timepoints is consistent with cartilage’s heterogeneous structure. Interstitial water in cartilage exists in two distinct compartments, namely intrafibrillar water (within collagen fibers) and extrafibrillar water that can move freely throughout the tissue under pressure gradients [49]. Multiple studies have reported that diffusion coefficients vary significantly with cartilage depth, with values 1.6 to 2.4 times greater in the surface zone as compared to the mid-zone [50], with diffusion coefficients up to tenfold higher in the superficial versus the middle zones [51]. Given that our microsphere size (0.58–0.82 µm) substantially exceeds the 0.05–0.06 µm pore size of the collagen type II fibrillar network in the superficial zone [5,6], particles are likely confined to larger extrafibrillar spaces. This size exclusion distinguishes our microspheres from smaller molecules (<0.1 µm) that can access intrafibrillar spaces [8,52].

Avidinated microspheres demonstrated visibly enhanced cartilage penetration (Figure 3E,F). The dense network of ECM components and the anionic charge of glycosaminoglycans limit the diffusion of delivery systems with negative charges [5,18]. Avidin’s tetrameric structure and high isoelectric point (pI ~ 10) confer a strong net positive charge at physiological pH, enabling electrostatic attraction to negatively charged glycosaminoglycans [19,45,53]. Single-timepoint (24 h) bi-exponential modeling yielded excellent fits with R^2^ = 0.850–0.913 (x-direction), compared to Fickian R^2^ = 0.598–0.847 (Table S1). Diffusion coefficient approximations of *D*_fast_ = 420.3–447.2 μm^2^/s and *D*_slow_ = 0.2060–0.2677 μm^2^/s were extracted, representing 40.6–47.3% fast and 52.7–59.4% slow transport fractions, respectively (Table 1). The higher proportion of fast transport than unmodified microspheres is consistent with avidin’s electrostatic interactions facilitating movement through the negatively charged matrix. Despite improved penetration depth, multi-timepoint validation failed with overall R^2^ values ranging from −1.0510 to 0.2942.

In contrast, PEGylated microspheres exhibited the deepest cartilage penetration (Figure 3E,G). While avidin was selected for charge-based interactions, PEGylation represents a complementary strategy through stealth properties. As PLGA is inherently hydrophobic, PEGylation creates a hydrophilic shell that improves microsphere dispersibility in the aqueous cartilage environment, reducing particle aggregation and enhancing interactions with the hydrated ECM [46,54]. Single-timepoint (24 h) bi-exponential modeling yielded better fits with R^2^ = 0.706–0.893 (x-direction) as compared to Fickian R^2^ = 0.656–0.766 (Table S1). Analysis showed diffusion coefficient approximations of *D*_fast_ = 414.2–448.4 μm^2^/s and *D*_slow_ = 0.30–0.42 μm^2^/s, with fast transport representing a notably higher fraction (40.9–48.8%) compared to unmodified microspheres. The higher *D*_slow_ values suggest that the PEG coating enhances transport even through slower, more constrained paths. Despite presenting the deepest penetration, multi-timepoint validation similarly failed with overall R^2^ values of −0.1109 to 0.3358 in both directions.

The disconnect between adequate single-timepoint fits and failed multi-timepoint validation across all three formulations indicates that microsphere transport into cartilage is fundamentally non-diffusive. The success of single-timepoint fitting reflects the mathematical flexibility of the bi-exponential function to fit individual concentration profiles rather than representing true physical diffusion. The observation that surface modifications dramatically enhance penetration depth (unmodified < avidinated < PEGylated) while failing to enable classical diffusive transport suggests that these modifications influence the spatial distribution and extent of microsphere penetration without fundamentally changing the transport mechanism. Avidin’s positive charge facilitates deeper initial penetration through electrostatic interactions, while PEG’s hydrophilic layer reduces protein adsorption and steric interactions with the dense matrix, enabling microspheres to navigate more deeply into extrafibrillar spaces. However, both formulations remain subject to size exclusion constraints that prevent access to intrafibrillar pathways.

### 3.3. Drug Encapsulation and In Vitro Release Kinetics

Hydrocortisone-17-butyrate (H-17-B) was selected as the model corticosteroid due to its enhanced lipophilicity and potency compared to standard hydrocortisone, providing approximately 10-fold greater anti-inflammatory potency than hydrocortisone (HC) and allowing for lower dosing requirements while maintaining therapeutic efficacy [55,56]. The butyrate esterification at the C-17 position increases the drug’s hydrophobic character, improving compatibility with the hydrophobic PLGA polymer matrix. In vitro drug release profiles of H-17-B at either 24 or 50 µg/mL for unmodified, avidinated, and PEGylated PLGA microspheres using the optimized emulsion conditions (5% (*w*/*v*) PVA, 25% sonication intensity, *M*_H-17-B_/*M*_PLGA_ = 0.3) were evaluated following United States Pharmacopeia (USP) Type II dissolution standards (i.e., 50 rpm and 37 °C bath temperature to simulate the temperature of the human body) in release medium (1× PBS, pH 7.4) [24].

**Figure 4:**
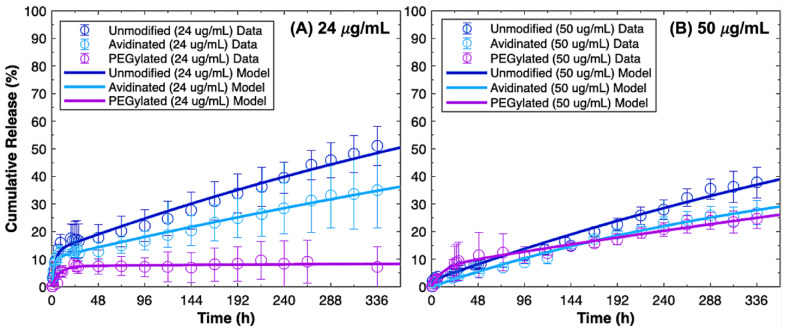
Cumulative release (%) of hydrocortisone-17-butyrate (H-17-B) and its hydrolysis product, hydrocortisone (HC), from PLGA microspheres at (**A**) C_0_ = 24 μg/mL and (**B**) C_0_ = 50 μg/mL. Data represent mean ± SD (*n* = 3).

All formulations exhibited biphasic release kinetics characterized by an initial burst release within the first 24–48 h, followed by a sustained release phase through Day 14 (Figure 4). Surface modification significantly influenced release kinetics. Both avidinated and PEGylated MSs exhibited slower overall release compared to unmodified MSs, with 34.91% and 7.1% cumulative release, respectively, by Day 14, versus 50.99% for unmodified particles at an initial H-17-B concentration of 24 µg/mL. Similar results were observed at the higher concentration of 50 µg/mL: 24.64% (PEGylated) < 29.72% (avidinated) < 37.75% (unmodified). This difference may be attributed to several mechanisms. The conjugation of hydrophilic avidin or PEG chains to the PLGA surface may create a barrier layer that sterically hinders water penetration, thereby slowing bulk erosion and hydrolytic degradation of the polymer matrix [22,57]. Donaldson et al. (2013) demonstrated quantitatively that biotinylation of PLGA microparticles slowed both drug desorption rates and effective drug diffusivity, consistent with the formation of a surface diffusion barrier [58]. The more pronounced effect observed with PEGylation compared to avidin conjugation suggests that PEG chains provide greater steric hindrance due to their flexible hydrophilic nature and ability to form extended brush conformations at the polymer–water interface [46].

**Figure 5:**
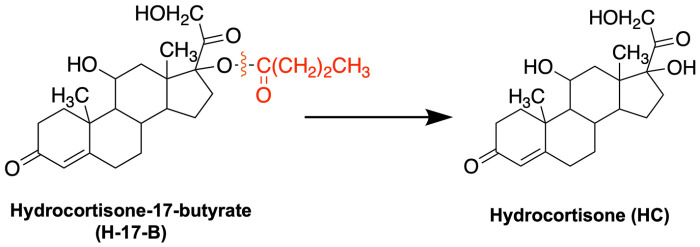
Ester hydrolysis reaction showing cleavage of the butyrate group (depicted in red) at the C-17 position.

**Figure 6:**
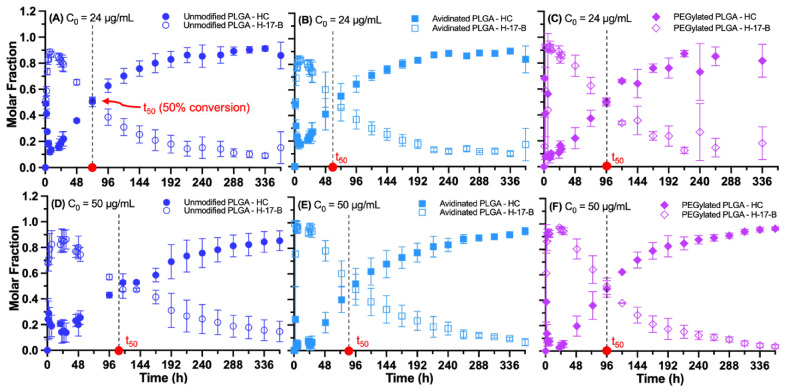
Molar conversion of H-17-B to HC via ester hydrolysis for (**A**,**D**) unmodified, (**B**,**E**) avidinated, and (**C**,**F**) PEGylated PLGA MSs. Data represent mean ± SD (*n* = 3).

Given the complex release behavior observed, the experimental data were fit to multiple kinetic models, including zero-order, first-order, Higuchi, Korsmeyer-Peppas, and bi-exponential models. The bi-exponential model provided the best fit for all formulations, with R^2^ values exceeding 0.8648 (Table S2). The bi-exponential model captures the biphasic nature of PLGA-based drug delivery [22,57]. The initial rapid phase (*k*_fast_) likely reflects surface desorption and drug dissolution near the particle surface, while the slower second phase (*k*_slow_) is consistent with polymer erosion-mediated drug liberation [57]. This dual-phase behavior is characteristic of hydrophobic drug release from PLGA and reflects the complex interplay between diffusion-controlled and erosion-controlled mechanisms. At both H-17-B concentrations, higher rapid-phase release was observed with unmodified microspheres as compared to modified microspheres (up to *k*_fast_ = 0.9992 h^−1^ with unmodified versus 0.4791 h^−1^ with modified microspheres) (Table 2). Analysis of ester hydrolysis kinetics revealed concentration-dependent drug degradation of unmodified microspheres (Figure 5). The transformation of H-17-B to less potent HC was quantified across 14 days by separately integrating HPLC peaks corresponding to each compound. The molar fraction of HC at each timepoint was calculated as [HC]/([H-17-B] + [HC]), providing a direct measure of ester hydrolysis progression. For unmodified microspheres, the hydrolysis rate was strongly concentration-dependent. At 24 µg/mL initial H-17-B concentration, the time to 50% molar transformation (*t*_50_) was approximately 72 h, whereas at 50 µg/mL, *t*_50_ was 120 h, representing an approximately 2-fold increase in transformation time. Similarly, for avidinated microspheres, *t*_50_ was approximately 60 h at 24 µg/mL and ~80 h at 50 µg/mL, an approximately 1.33-fold increase in transformation time. By 96 h, the molar fraction of HC reached 0.628 at 24 µg/mL compared to only 0.429 at 50 µg/mL for unmodified MSs and 0.646 and 0.523 HC molar fractions at 24 µg/mL with avidinated MSs, respectively (Figure 6). This concentration dependence suggests that the hydrolysis mechanism may involve saturable processes or that higher local drug concentrations provide some degree of protection against transformation, possibly through altered microenvironment pH or reduced water penetration into drug-rich regions. In striking contrast, PEGylated microspheres showed *t*_50_ values of 96 h at both concentrations (Figure 6C,F), with similar HC molar fractions at 96 h for both H-17-B (0.505 at 24 µg/mL and 0.490 at 50 µg/mL). By Day 14, HC conversion was 91.3% for unmodified microspheres at 24 μg/mL (representing 46.6% loss in theoretical anti-inflammatory potency), 77.4% for avidinated (22.6% potency loss), and 50.5% for PEGylated formulations (3.6% potency loss).

The concentration-dependent transformation kinetics observed for unmodified PLGA microspheres, which exhibited faster hydrolysis at lower drug concentrations, suggest a mechanism beyond simple first-order chemical hydrolysis. Higher drug loading may alter local pH through accelerated PLGA degradation and accumulation of acidic degradation products (lactic and glycolic acid) [57], create more hydrophobic microdomains that limit water penetration, or result in different physical states of the drug (crystalline vs. amorphous) within the polymer matrix, with crystalline forms potentially being more resistant to hydrolysis [59]. Surface-modified microspheres, particularly PEGylated MSs, exhibit concentration-independent transformation kinetics, suggesting that avidin and PEG conjugation fundamentally alter the drug release microenvironment. The hydrophilic PEG shell may create a buffering layer that maintains consistent local pH, whereas the avidin coating may provide steric protection to stabilize the ester bond. These stabilization effects represent an unexpected benefit beyond enhancing cartilage penetration and retention.

### 3.4. Integrated Discussion

Integration of fabrication control, cartilage transport characterization, and drug release analysis reveals critical design trade-offs for optimizing PLGA microsphere formulations for intra-articular corticosteroid delivery. This study demonstrates how surface modifications simultaneously affect multiple performance parameters, particularly cartilage penetration depth, drug release kinetics, and drug chemical stability. Fabrication parameter optimization demonstrated precise control over particle size (0.58–0.98 μm) and surface charge through systematic variation in PVA concentration and sonication intensity. Surface functionalization with avidin and PEG successfully altered surface chemistry while maintaining acceptable PDI (<0.3) and near-neutral zeta potentials, establishing three distinct formulations suitable for tissue penetration applications.

Cartilage penetration studies revealed the striking hierarchy: unmodified < avidinated < PEGylated, representing 2–3× improvement for PEGylated formulations. Mathematical modeling demonstrated that bi-exponential models provide superior fits (R^2^ > 0.706–0.999) compared to Fickian models (R^2^ = 0.040–0.886) at the 24 h comparison, explicitly accounting for cartilage’s heterogeneous dual-pathway structure. However, multi-timepoint validation revealed non-diffusive transport for all formulations, indicating fundamental size exclusion constraints from the collagen fibrillar network (0.05–0.06 μm pores). The observed contradiction, namely enhanced penetration without classical diffusion, can be reconciled by recognizing that surface modifications alter the spatial distribution and extent of microsphere penetration through distinct mechanisms. Avidin’s positive charge (pI ~ 10) provides electrostatic interactions with anionic glycosaminoglycans (*K*_D_ ~ 150 μM, *N*_T_ ~ 2920 μM), increasing the fast transport fraction to 31.3–51.3% versus 17.1–38.1% for unmodified particles [19,45,53]. In comparison, PEG’s hydrophilic layer reduces protein adsorption and steric interactions, achieving a 38.1–48.0% fast transport fraction with elevated *D*_slow_ values (0.1224–0.4346 vs. 0.1224–0.1941 μm^2^/s with unmodified microspheres), demonstrating enhanced transport through both fast and slow pathways.

Drug release studies demonstrated that these same surface modifications dramatically alter release kinetics and drug stability. Surface-modified microspheres exhibited substantially slower release: by Day 14, PEGylated microspheres released only 7.1% compared to 50.99% for unmodified particles at 24 μg/mL. This sustained release profile addresses a critical limitation of conventional intra-articular corticosteroid injections, which suffer from rapid clearance (half-lives of 4–8 h), necessitate frequent dosing, and can rapidly accelerate ester hydrolysis due to exposure to synovial fluid, which contains esterases and proteases and has neutral pH conditions [60,61]. Importantly, surface modifications also stabilized H-17-B against ester hydrolysis, representing an unexpected benefit beyond improved tissue penetration. Unmodified microspheres exhibited 46.6% potency loss by Day 14, while PEGylated formulations showed only 3.6% loss. This stabilization likely results from the hydrophilic surface coating creating a buffering layer that maintains consistent microenvironment pH and reduces water penetration, protecting the ester bond from hydrolysis. The convergence of these findings establishes fundamental design principles: surface chemistry optimization simultaneously controls tissue penetration behavior through modulation of electrostatic and steric interactions, release kinetics through alteration of water penetration and polymer degradation rates, and drug chemical stability through microenvironment control. While size-based transport barriers fundamentally limit absolute penetration depth regardless of surface modification, the combination of enhanced extrafibrillar penetration, sustained release kinetics, and preserved drug potency positions surface-modified PLGA microspheres, especially PEGylated formulations, as promising candidates for prolonged intra-articular corticosteroid therapy.

## 4. Conclusions

This work establishes critical design principles for optimizing PLGA microsphere formulations for intra-articular corticosteroid delivery by systematically investigating fabrication parameters, surface modifications, and drug release mechanisms. Fabrication optimization demonstrated that particle size (0.58–0.98 μm) and polydispersity could be precisely controlled through variation in PVA concentration (1–5% *w*/*v*) and sonication intensity (20–30%), with higher PVA concentrations and sonication intensities producing smaller, more uniform particles suitable for tissue penetration applications. Surface functionalization with avidin and PEG successfully altered surface chemistry and dramatically enhanced cartilage penetration, with PEGylated microspheres achieving 2–3× deeper penetration compared to unmodified formulations.

Mathematical analysis of cartilage penetration revealed that bi-exponential diffusion models provide superior fits to depth-dependent concentration profiles compared to classical Fickian models (R^2^ > 0.706 vs. 0.444–0.886), explicitly accounting for cartilage’s heterogeneous dual-pathway structure comprising fast extrafibrillar and slow intrafibrillar transport. However, multi-timepoint validation demonstrated that microsphere transport, regardless of surface modification, does not follow classical diffusion kinetics, as diffusion coefficients could not be maintained constant over time. This fundamental non-diffusive behavior indicates that microsphere penetration is constrained by size exclusion from the collagen fibrillar network (0.05–0.06 μm pore size) regardless of surface chemistry. Nevertheless, surface modifications substantially influence penetration depth and spatial distribution: avidin’s positive charge (pI ~ 10) provides electrostatic interactions with anionic glycosaminoglycans, while PEG’s hydrophilic coating reduces matrix interactions through stealth properties, enabling microspheres to navigate deeper into extrafibrillar spaces.

Drug release studies revealed biphasic kinetics best described by bi-exponential models (R^2^ > 0.8648), with surface modifications substantially slowing release rates through the creation of hydrophilic barrier layers that hinder water penetration and polymer degradation. By Day 14, PEGylated microspheres released only 7.1% compared to 50.99% for unmodified particles at 24 μg/mL initial concentration, addressing the critical limitation of conventional intra-articular corticosteroid injections, which suffer from rapid clearance (half-lives of 4–8 h) and necessitate frequent dosing. Importantly, surface modifications also stabilized H-17-B against ester hydrolysis during release, representing an unexpected benefit beyond the intended goals of improving tissue penetration. Unmodified microspheres exhibited concentration-dependent transformation kinetics with 91.3% conversion to the less potent hydrocortisone by Day 14 (46.6% potency loss), while PEGylated formulations showed concentration-independent stability with only 50.5% conversion (3.6% potency loss). This stabilization likely results from reduced water penetration through the hydrophilic surface coating and maintenance of consistent microenvironment pH, protecting the ester bond from hydrolysis. The preservation of drug potency throughout the release period could translate to improved therapeutic outcomes, lower required doses, and less frequent injections in clinical applications.

Integration of these findings reveals that surface chemistry optimization simultaneously controls tissue penetration behavior, release kinetics, and drug chemical stability. The dual benefits of surface functionalization, specifically enhanced penetration through reduced matrix interactions combined with improved drug stability through microenvironment control, expand the design space for controlled-release formulations. While size-based transport barriers limit absolute penetration depth regardless of surface modification, the combination of sustained release kinetics (minimizing burst release and extending drug residence time), enhanced cartilage penetration (particularly for PEGylated formulations), and preserved drug potency (maintaining therapeutic efficacy throughout release) positions surface-modified PLGA microspheres as promising candidates for prolonged intra-articular corticosteroid therapy in osteoarthritis treatment. Future studies will validate these findings in preclinical animal models of osteoarthritis to assess whether the enhanced penetration, sustained release, and preserved potency observed in vitro translate to improved therapeutic outcomes in vivo. Such validation will establish dosing regimens and safety profiles necessary for clinical translation. This framework establishes a comprehensive approach for rational design of PLGA-based intra-articular delivery systems, demonstrating that thoughtful surface chemistry design achieves synergistic benefits, positioning surface-modified PLGA microspheres as a platform technology for treating osteoarthritis and potentially other joint diseases requiring sustained intra-articular drug delivery.

## Figures and Tables

**Table 1:** Bi-exponential diffusion approximation parameters for fluorescently labeled unmodified, fluorescently avidinated, and PEGylated PLGA microspheres. Goodness-of-fit (R^2^) values for bi-exponential mathematical models fitted to 1, 2, 5, 18, and 24 h diffusion into bovine osteochondral plugs. Data represent mean ± SD (*n* = 3).

Direction	Intensity	*D*_fast_ (µm^2^/s)	*D*_slow_ (µm^2^/s)	*f*_fast_ (%)	Overall R^2^	R^2^				
						1 h	2 h	5 h	18 h	24 h
Unmodified (x-direction)	20%	333.9	0.1553	25.9	−0.2094	−32.44	−2.926	−0.533	0.148	−4.422
	25%	381.0	0.1371	38.1	0.3101	−0.266	0.458	0.882	0.489	0.045
	30%	362.0	0.1243	31.3	0.1662	0.062	−70.27	0.680	0.163	0.057
Unmodified (y-direction)	20%	349.9	0.1941	17.1	−2.3579	−5.453	−10.47	−3.149	0.146	0.186
	25%	500	0.1224	37.5	−0.7794	−10.02	−2.060	0.749	−0.245	−0.834
	30%	360.5	0.5520	28.4	0.0265	−2.464	−0.613	0.693	0.214	0.264
Avidinated (x-direction)	20%	447.2	0.2060	47.5	−1.0510	−11.49	−0.610	0.722	−34.51	−0.012
	25%	331.5	0.2677	31.3	−0.8240	−8.133	−24.33	0.208	0.427	0.197
	30%	420.3	0.2521	40.6	0.2942	0.184	−1.420	0.450	0.657	0.386
Avidinated (y-direction)	20%	500.0	0.1238	40.8	−0.4848	−10.97	−5.473	−0.924	−3.841	0.196
	25%	500.0	0.1227	39.6	−0.6422	−1.855	−4.724	0.040	−5.464	−0.197
	30%	406.1	0.2988	51.3	0.2727	−37.67	0.246	0.291	−0.108	0.231
PEGylated (x-direction)	20%	414.2	0.2507	44.5	0.3358	−10.11	0.747	0.689	−0.203	−2.246
	25%	439.7	0.2638	38.4	0.2037	0.714	−22.62	−6.640	0.566	0.358
	30%	448.4	0.4346	43.1	0.1537	−42.28	0.139	0.926	−0.011	0.351
PEGylated (y-direction)	20%	500.0	0.1224	38.1	−0.0116	0.057	−0.690	0.694	−1.216	−0.237
	25%	456.2	0.1234	45.9	0.0225	−1.997	0.361	−0.406	−4.015	0.795
	30%	439.6	0.2211	48.0	−0.1109	−2.869	−1.774	0.184	−0.075	0.307

**Table 2:** Bi-exponential model fits for hydrocortisone-17-butyrate (H-17-B) release from PLGA microspheres. Release profiles for unmodified, avidinated, and PEGylated formulations over 14 days with fitted bi-exponential curves. Data represent mean ± SD (*n* = 3).

Formulation	H-17-B Concentration	*φ*_fast_ (%)	*k*_fast_ (h^−1^)	*φ*_slow_ (%)	*k*_slow_ (h^−1^)	Total (%)	R^2^
Unmodified	24 µg/mL	12.92	0.2765	100	0.0013	112.92	0.9863
Avidinated	24 µg/mL	10.39	0.4791	100	0.0008	110.39	0.9915
PEGylated	24 µg/mL	7.44	0.1263	1.28	0.0032	8.72	0.8648
Unmodified	50 µg/mL	2.24	0.9992	100	0.0013	102.23	0.9893
Avidinated	50 µg/mL	25.21	0.0024	25.30	0.0024	50.51	0.9916
PEGylated	50 µg/mL	7.35	0.0851	99.93	0.0006	107.28	0.9880

## Data Availability

The original contributions presented in this study are included in the article/supplementary material. Further inquiries can be directed to the corresponding author.

## Supplementary Materials

The following supporting information can be downloaded at https://www.mdpi.com/article/10.3390/pharmaceutics18070893/s1, Table S1. Kinetic model comparison for fluorescently labeled unmodified, avidinated, and PEGylated PLGA microspheres. Goodness-of-fit (R^2^) values for six mathematical models fitted to 24 h diffusion into bovine osteochondral plugs. Data represent mean ± SD (*n* = 3). Table S2. Kinetic model comparison for hydrocortisone-17-butyrate (H-17-B) from PLGA microspheres. Goodness-of-fit (R^2^) values for five mathematical models fitted to 14-day release data from unmodified, avidinated, and PEGylated formulations at two initial drug concentrations (24 and 50 µg/mL). Data represent mean ± SD (*n* = 3).
