# Supplementary material for: Engineering Poly(Lactic-co-Glycolic Acid) (PLGA)-Based Microspheres for Controlled Corticosteroid Delivery in Intra-Articular Cartilage
Source: Pharmaceutics. 2026 Jul 21;18(7):893. doi: 10.3390/pharmaceutics18070893 (PMC13415598; doi:10.3390/pharmaceutics18070893)
Supplement: Supplementary file 1 [file pharmaceutics-18-00893-s001.zip › pharmaceutics-4198150 - SM updated.pdf]

Supporting Information For:

Engineering Poly(Lactic-co-Glycolic Acid) (PLGA)-Based Microspheres for Controlled Corticosteroid Delivery in Intra-Articular Cartilage

Pamela Rose V. Samonte[†] and Noelle K. Comolli[†*]

[†]Department of Chemical and Biological Engineering, Villanova University, 800 E. Lancaster Ave., Villanova, PA 19085

*Corresponding author at: Department of Chemical and Biological Engineering, Villanova University, 800 E. Lancaster Ave., Villanova, PA 19085, USA

E-mail address: noelle.comolli@villanova.edu (Noelle Comolli)

Phone No.: +1-610-519-7134

Table S1. Kinetic model comparison for fluorescently labeled unmodified, avidinated, and PEGylated PLGA microspheres. Goodness-of-fit (R^2) values for six mathematical models fitted to 24-h diffusion into bovine osteochondral plugs. Data represent mean ± SD (*n* = 3).

Formulation	Sonication Intensity	Diffusion model					
		**Fickian**	**Bi-exponential**	**Anomalous**	**Stretched exponential**	**Diffusion +Decay**	**Conv+Diff**
Unmodified (x-direction)	20%	0.886	0.986	0.915	-1.992	0.876	0.471
	25%	0.764	0.945	0.777	-1.235	0.801	0.772
	30%	0.610	0.978	0.826	-0.579	0.618	0.750
Unmodified (y-direction)	20%	0.815	0.999	0.952	-0.444	0.802	0.792
	25%	0.553	0.989	0.729	-0.454	0.636	0.680
	30%	0.689	0.999	0.993	-0.006	0.672	0.917
Avidinated (x-direction)	20%	0.598	0.850	0.607	-0.080	0.706	0.631
	25%	0.731	0.952	0.850	-0.262	0.877	0.747
	30%	0.847	0.913	0.854	-0.382	0.891	0.850
Avidinated (y-direction)	20%	0.186	0.965	0.186	-1.494	0.306	0.477
	25%	0.040	0.894	-0.003	-1.621	-0.029	0.429
	30%	0.759	0.869	0.762	-0.323	0.812	0.756
PEGylated (x-direction)	20%	0.656	0.706	0.656	0.319	0.488	0.629
	25%	0.675	0.893	0.673	-1.082	0.752	0.721
	30%	0.766	0.822	0.774	-0.330	0.832	0.779
PEGylated (y-direction)	20%	0.648	0.860	0.650	-1.250	0.700	0.693
	25%	0.444	0.989	0.439	-0.516	0.634	0.649
	30%	0.808	0.830	0.809	-0.327	0.855	0.817

Table S2. Kinetic model comparison for hydrocortisone-17-butyrate (H-17-B) from PLGA microspheres. Goodness-of-fit (R^2) values for five mathematical models fitted to 14-day release data from unmodified, avidinated, and PEGylated formulations at two initial drug concentrations (24 and 50 µg/mL). Data represent mean ± SD (n = 3).

Formulation	H-17-B Concentration	Diffusion model				
		Fickian	Higuchi	Bi-exponential	Weibull	First-order
Unmodified	24 µg/mL	0.5524	0.9373	0.9863	0.9399	0.8099
Avidinated	24 µg/mL	0.5152	0.8782	0.9915	0.9234	0.7128
PEGylated	24 µg/mL	-7.5914	0.1086	0.8648	0.8614	0.8611
Unmodified	50 µg/mL	0.2999	0.9136	0.9893	0.9788	0.9757
Avidinated	50 µg/mL	0.4569	0.9303	0.9916	0.9916	0.9916
PEGylated	50 µg/mL	0.4355	0.9751	0.9880	0.9729	0.9247
